# Survey of workplace stressors among Hungarian elite athletes

**DOI:** 10.3389/fpsyg.2025.1637069

**Published:** 2025-08-18

**Authors:** Viktória Resperger, Anikó Kapros, Tamás Berki

**Affiliations:** ^1^Department of Psychology and Sport Psychology, Institute of Economic and Social Sciences, Hungarian University of Sports Science, Budapest, Hungary; ^2^Doctoral School of Military Sciences, Ludovika University of Public Service, Budapest, Hungary; ^3^Department of Physical Education Theory and Methodology, Hungarian University of Sports Science, Budapest, Hungary

**Keywords:** workplace stressors, athletes, stress factors, individual sport, team sports

## Abstract

**Introduction:**

People working in sports, particularly athletes, are vulnerable to workplace stressors. These stressors can arise from various factors and significantly impact an individual’s mental, physical, and emotional well-being. This ongoing research maps out the workplace stressors affecting professional Hungarian athletes involved in competitive sports. The study identifies work-related factors that may influence athletes’ stress levels. It highlights the parallels between traditional workplace stressors and stressors that affect athletes and shows these stressors across different genders, types of sports, and competition levels. The results also compare the stressors faced by various kinds of sports (such as individual versus team sports).

**Methods:**

The research was conducted in two stages. A questionnaire prepared as a starting point was designed for a quantitative data collection method using questionnaires. A qualitative preparatory phase was included to refine the questionnaire. The study emphasizes the significance of work-related stress amongst athletes by explicitly examining workplace stressors in sports using an established model.

**Results:**

The findings show ten factors and three second-order factors for measuring work-related stress among athletes. The research also identifies differences between how male and female athletes perceive work-related stress. Furthermore, the results indicate that individual athletes experience higher work-related stress levels than team sports.

**Discussion:**

Our research highlights the importance of recognizing and addressing the various stressors athletes face throughout their careers.

## Introduction

Those working in sport, whether they are athletes, coaches, sports administrators, or support staff, are particularly exposed to the challenges caused by workplace stress (Fletcher and Arnold, 2017). These stressors can arise from a variety of factors and have a significant impact on the mental, physical, and emotional wellbeing of individuals (Rice et al., 2016). Workplace stressors in sports can include a bad trainer, discomfort from difficult working conditions (too cold, too hot), unsanitary changing rooms, constant travel, and disruption of circadian rhythms, among other causes. However, it is not clear which of these has an impact on athletic performance and mental state. Athletes have the same goals in mind: how to achieve peak performance, how to win the next competition, and how to achieve their own personal performance. To achieve these goals, there are many stressful factors that need to be taken into account, including traveling, organization, maintaining a diet, and communication problems (Tossici et al., 2024). These, and other stressors, can at times lead to burnout syndrome in the short term, from which it is very difficult to bring the athlete back to peak performance (Woods et al., 2022). Moreover, if we are aware of the stressors that the athlete finds most difficult to cope with, we can help them to manage them more effectively. In occupational psychology, the study of workplace stressors has a long tradition in the literature (e.g., Goh et al., 2015; Maudgalya et al., 2006). However, professional athletes, whose actual work is sport, encounter different types of workplace stressors that are rarely studied. In our research, we map the workplace stress factors affecting Hungarian elite athletes, both active and retired, in sports.

### Workplace stressors

Many people understand workplace stress to mean a conflict between managers and subordinates, employers and employees (Woodman and Hardy, 2001). However, workplace stress covers a much broader spectrum. In the case of different stressors, it is very important how predictable or influenceable they are for the individual. Numerous studies have shown that if we can prepare for something and influence it, it will cause us much less stress than things that are unpredictable (Nolen-Hoeksema et al., 2009). The same applies to stressors at work: some can be planned in advance (e.g., I have to commute to work every day), but there are others, such as interpersonal conflicts and the work environment (noise, temperature), over which we have less control (Klein, 2004; Juhász, 2002). The impact of workplace stress on employee motivation, performance, wellbeing, and productivity is one of the most significant challenges facing organizations. Research has shown that employees who experience high levels of stress perform less and are more likely to suffer from serious physical and mental health problems in the long term (Muñoz et al., 2022).

Workplace stress often arises from excessive expectations, deadlines, competitive situations, or a lack of balance between work and private life. Stressors in sport, such as competitive pressure, performance pressure, or the risk of injury (Lehrer et al., 2007), can also place a significant psychological burden on athletes. However, little research has been conducted into the impact of other stressors affecting athletes, known as athletic workplace stressors, on athletic performance. The literature on workplace stressors covers a wide spectrum, but the classification by Juhász (2002) presented below seems to be the most detailed, which is why we chose it as the basis for our research, as these are even less of a focus in sports.

The stressors associated with the job vary widely: meeting deadlines and excessive or insufficient workloads in terms of quantity or quality can be significant sources of stress in the life of an employee (Cooper and Davidson, 1987). An equally significant stressor is the need to keep pace with technological changes, where generational differences can also be observed, so it is worth providing them with different motivation systems (Resperger and Géczi, 2021). Although it is a relatively new concept, we also need to address the issue of technostress. We need to keep pace with the latest technological changes in order to improve our position in the labor market. Keeping pace is stressful, as it often involves a complex learning process (Szikora, 2023).

Work-related stressors are among the most significant stressors (Juhász, 2002). Working or playing sports in extreme weather conditions all day long (e.g., ice hockey players, tennis players, runners, etc.) and performing well amid constant noise is extremely difficult. Juhász (2002) highlights that stressors related to roles within the organization can come from three main sources: they can be individual conflicts, which may arise, for example, from a lack of individual career prospects, but they can also occur at the group level (e.g., ostracism) or at the organizational level, when we feel insecure about our job or work in a poor workplace atmosphere. Juhász (2002) gives several examples of stressors outside the organization, one being when daily commuting causes problems because the individual lives far from the workplace and has to spend long hours traveling.

In sports, we have recently encountered the fifth major group of workplace stressors. These include stressors specific to certain groups, such as women (Juhász, 2002). In recent days, countless articles have appeared about the humiliation of Italian female gymnasts and about figure skater Marina Kielmann and the abuse she suffered at the hands of her team doctor (Kiss, 2025). There is considerable evidence that women report greater fear and are more likely to develop anxiety disorders than men (McLean and Anderson, 2009). Further research examining differences between women and men focused on social anxiety. Although this area has not yet been fully explored, research to date suggests that gender differences can also be found in this area (Caballo et al., 2014). The article by Medaris (2023) draws attention to the gender differences in stress, existing from additional domestic expectations toward women, and also societal pressures.

### Stress in sports

The influential work by Fletcher et al. (2012) indicates that organizational stress plays a significant role in athlete performance, which are directly linked to the act of competing such as anxiety before a match, fear of underperforming, or pressure to win. This work has paved the way for further studies. The authors argue that understanding stress in sport needs to expand beyond competitive anxiety to include the considerable influence of organizational factors. Numerous international studies have shown that athletes often experience anxiety, melancholy, and even depression (Grobler et al., 2022). The findings of Mellalieu et al. (2009) demonstrate that, in addition to performance-related stress, athletes also have to face work-related stressors, which stem from the broader occupational context in which athletes operate. Both can affect the overall stress experienced by athletes.

A lack of performance level, loss of enthusiasm, and even burnout can occur when someone is faced with unmanageable stress (Jiménez-Picón et al., 2021). Athletes with greater mental toughness often have the ability to perceive stressors as less severe, leading them to a more positive mindset and better stress response (Cowden, 2016). According to the study of Nuetzel (2023), elite athletes face multiple sources of stress. It can arise from competition, organizational demands, and personal issues that can all add to the risk of mental health problems. The study of Hamlin et al. (2019) indicates that workload changes are consistent with higher stress levels among the athletes tested. Furthermore, they found that changes occurring during the pre-season periods have a greater effect on rising stress levels. Den Hartigh et al. (2024) highlight that stress management, alongside resilience, are both important factors in helping athletes bounce back after facing challenges. Overall, various types of work-related stress have an impact on a person’s wellbeing (e.g., Parry et al., 2025).

### Aims of the study

In our research, we examine workplace stressors specifically in sports, using our established model of workplace stressors. Being an athlete is a unique form of employment; athletes do not follow a typical 40-h work week from 8 a.m. to 4 p.m., nor do they usually work at their employer’s premises. Our goal is to identify factors that may influence athletes’ stress levels, in line with previous models (Juhász, 2002). Best of our knowledge this is the first empirical analysis of the model in sport context. Although Juhász’s (2002) model was originally developed for general workplace settings, its comprehensive categorization of stressors makes it highly adaptable to the elite sport context. This adaptability is particularly valuable given the occupational nature of professional athletic careers. Additionally, we aim to determine whether stressors that are unique to athletes can be identified, and if there are any gender differences in how these stressors are experienced among athletes. Finally, we want to compare the stressors faced by different types of sports (such as individual versus team sports) and explore the varying experiences of competitors.

## Methods

### Procedure

The research was conducted in two stages between October 2024 and April 2025. The questionnaire prepared as a starting point was designed for a quantitative data collection method using questionnaires. In order to refine the questionnaire, a qualitative preparatory phase was included. This phase consisted of interviews with 10 former elite athletes from 10 different sports. They were asked to reflect on the stressful situations they had experienced during their sporting careers. The final questionnaire was refined and supplemented using the results of the interviews. The survey was compiled using SurveyMonkey questionnaire software and distributed online via the same platform. The questionnaire was widely distributed among athletes, sports organizations, and university students. It took approximately 15–20 min to complete. The questionnaire was anonymous. The study was conducted in accordance with the Declaration of Helsinki (World Medical Association, 2013), and the guidelines of the Ethics Committee at the Hungarian University of Sports Science were followed (Ethical number: MTSE-OKE-KEB/04/2023).

### Participants

The 10 interviewees included active competitors, athletes participating in national championships, federation leaders, coaches, Olympians, and former Olympic champions. The questionnaire was designed specifically for Hungarian competitive athletes. The minimum age for completing the questionnaire was 18. A total of 319 responses were recorded during the data collection process. Unfortunately, we had to exclude 48 responses from the analysis due to incomplete data, which slightly reduced our final sample size. A total of 271 responses were usable during data processing. Among the respondents, 156 were male and 115 were female. Regarding sport type, 44% (*n* = 120) were individual athletes, while 56% (*n* = 151) took part in team sports.

### Interviews and the questionnaire

The methodology used enabled a comprehensive and reliable mapping of athletes’ life circumstances, work situations, and stressors, while the qualitative preparation process ensured the practical relevance of the questionnaire. The interviews were used in the initial stage of the research to create the questionnaire and ensure its relevance to athletes (Falus and Ollé, 2017, p. 61). Subsequently, active and retired athletes at various competitive levels took part in developing the questionnaire, sharing their experiences and offering suggestions after reviewing all the questions. These interviews lasted an average of 60–80 min and were conducted in person. Based on the athletes’ feedback and their comments, we refined the structure and content of the questionnaire in some areas. In developing the final questionnaire, we considered all the suggestions, additions, and professional comments we received. During the interviews and evaluations related to the questionnaire, it was important to ensure that both genders and both individual and team athletes were represented. Additionally, we involved coaches working with successful athletes in both individual and team sports in the research to ensure the questionnaire covered a wider range of sports. Before finalizing the questionnaire, we conducted a pilot test to gather additional practical feedback from respondents, which enhanced the reliability and validity of the tool.

In the questionnaire used, questions about stressors were grouped according to the classification of Juhász’s (2002) workplace stressors. The research covered the main categories listed in Table 1, such categories as task-related stressors, work environment stressors (physical environment), stressors related to roles within the organization, stressors occurring outside the organization, and stressors for specific layers.

**Table 1:** Categories of workplace stressors adapted from Juhász (2002, pp. 7–19).

Main category: Workplace stressors	Subcategories
Task-related stressors (missing deadlines, overtime, mistakes, etc.)	Quantitative or qualitative overloading or underloading
	Working conditions
	Changes in the work
	Keeping pace with rapid technological change, Technostress
Work environment stressors	- noise, - heat, - lighting, - air quality, etc.
Stressors related to roles within the organization	*At the individual level* - role ambiguity - role conflict - too much or too little responsibility for employees - career development
	*At a group level* - lack of cohesion - lack of good working relationships - relationship with superiors/subordinates
	*At an organizational level* - organizational climate - management styles - control systems - technology and its change - excessively low pay - job insecurity
Stressors outside the organization	- family relationships, financial problems - difficulties in balancing family and work roles - personal beliefs, convictions - frequent relocation - transportation to the workplace
Stressors for specific layers	- women (harassment at work, balancing work and family roles, gender inequality in the labor market) - physical workers (uncertainty, lack of understanding) - mental workers (family problems, interpersonal relationships) - stressors for leaders (e.g., stressors for middle managers) - subordinates’ stressors (e.g., lack of control)

The first part of the questionnaire contained demographic questions. The respondents were asked to provide information about their gender, age, and current place of residence in a closed, multiple-choice format. This was followed by a question about their sport and their current practices: they could choose from a predefined list or enter a free-text response if their sport was not included. The following questions concerned the results and their current relationship with sport.

The second part of the questionnaire contained a total of 66 statements relating to stressor categories. (e.g., *I find it stressful to deal with conflicts with my coach*.), which was based on our inital stage. For clarity, the statements were grouped and presented in six separate tables. Each statement was evaluated using a 6-point Likert scale, with the following answer options: “not at all characteristic,” “least characteristic,” “somewhat characteristic,” “moderately characteristic,” “usually characteristic,” and “always characteristic.”

### Statistical analysis

In addition to descriptive statistics, Principal Component Analysis (PCA) was conducted to identify the underlying factors within our 66-item questionnaire. Following the Kaiser criterion, factors with eigenvalues greater than 1 were retained for further analysis. The internal consistency of these factors was assessed using Cronbach’s alpha. To examine the bidirectional relationships among the identified factors, Pearson correlation coefficients were calculated. Subsequently, a Structural Equation Model (SEM) was employed to explore additional latent variables that could not be captured by the previous methods. In our SEM model, PCA results were used to build our model, and we identified second-order factors that were in line with theoretical models. Widely used fit indices were used to test our model fit, which included hi-square (x^2^), relative chi-square divided by the degree of freedom (CMIN/d.f), root mean square error of approximation (RMSEA), a non-normed fit index called the Tucker–Lewis index (TLI), comparative fit index (CFI), and standardized root mean square residual (SRMR). Finally, a one-way analysis of variance (ANOVA) was performed to investigate the effects of gender and various sport-related variables (e.g., sport type, team vs. individual sports, and competition level). At first step of ANOVA Levene’s test were used to test homogeneity of the variance, which were not violated in all cases, therefore Tukey’s *post hoc* were conducted for post-hoc comparison. All analysis were conducted with Jamovi 2.6.

## Results

Principal component analysis with varimax rotation was employed to examine the factor structure of our scale (see Table 2). A total of 52 items were included in this study, and these items were categorized into 10 distinct factors. The factors accounted for 65.72% of the variance and the Kaiser-Mayer-Olkin (KMO) index showed a 0.90 value. The first factor consisted of 9 items, with an eigenvalue of 5.58, accounting for 10.73% of the variance. This factor addressed stress perceived by peers and teammates, and was named “Athlete’s subordinates’ stressors.” The second factor had an eigenvalue of 4.89, explained 9.41% of the variance, and included seven items. It was named “Stressors outside of sports” as it pertained to stressors outside of sports. The third factor, with an eigenvalue of 4.82, explained 9.27% of the variance and included six items related to administrative stress that the athletes need to deal with on their own. We named this factor Individual stressors within the organization. The next factor included five items with an eigenvalue of 3.48 and explained 6.70% of the variance. It was named “Stressors from leader responsibilities” highlighting personal decisions regarding career continuation and team structure. The fifth factor, named “Sports conditions” had an eigenvalue of 3.09 and accounted for 5.94% of the variance. This factor contained questions about competition and equipment quality. The following factor was titled “Team stressors within the organization” with an eigenvalue of 2.85 and explained 5.47% of the variance. It included items concerning stressors from teammates. The seventh factor, which consisted of only three items related to workload and stress, was named “Overloading from sports.” This factor had an eigenvalue of 2.31 and accounted for 4.43% of the variance. The eighth factor explained 4.12% of the variance with an eigenvalue of 2.14 and included four items pertaining to changes in the sports environment, thus it was named “Changes in sports.” The next factor, called “Sports environment stressors” included items about adapting to the new sporting environment. It had an eigenvalue of 1.90 and explained 3.66% of the variance. Lastly, we named the final factor “Management stressors within the organization.” This factor included three items related to stress that individuals cannot influence and had an eigenvalue of 1.68, accounting for 3.23% of the variance. Additionally, there was one extra factor with a single item, which had an eigenvalue of 1.43 and explained 2.75% of the variance; however, we decided not to include it in further analysis.

**Table 2:** Final structure of our scale.

Items	Factors										
	1	2	3	4	5	6	7	8	9	10	11
Q38	0.74										
Q39	0.73										
Q42	0.71										
Q65	0.68										
Q40	0.67		0.40								
Q43	0.55										
Q66	0.54										
Q64	0.54										0.42
Q45	0.46					0.45					
Q51		0.77									
Q52		0.74									
Q50		0.66									
Q56		0.65									
Q54		0.65									
Q55		0.57									
Q53		0.51									
Q28			0.83								
Q27			0.82								
Q29			0.66								
Q41	0.51		0.64								
Q26			0.56								
Q30			0.55								
Q62				0.83							
Q63				0.82							
Q61				0.67							
Q37				0.51							
Q59		0.46		0.47							
Q10					0.78						
Q9					0.75						
Q11					0.58					0.44	
Q1					0.51						
Q12					0.48						
Q32						0.71					
Q31						0.67					
Q35						0.56					
Q34			0.47			0.50					
Q33						0.47					
Q3							0.71				
Q2							0.69				
Q7		0.44					0.49				
Q14								0.58			
Q21								0.58			
Q44								0.57			
Q20								0.45			
Q23			0.40						0.70		
Q22			0.45						0.65		
Q24									0.53		
Q6									0.35		
Q46										0.54	
Q47										0.49	
Q19							0.40			0.42	
Q60											0.63
Eigenvalue	5.58	4.89	4.82	3.48	3.09	2.85	2.31	2.14	1.90	1.68	1.43
% of variance	10.73	9.41	9.27	6.70	5.94	5.47	4.43	4.12	3.66	3.23	2.75

Factor loading higher than 0.04 were included in the analysis.

Cronbach’s alpha values and correlations of the factors are presented in Table 3 of the extracted factors. The mean scores range from 1.91 to 3.00, indicating that respondents experienced low to moderate levels of stress across different domains. The highest mean score was observed for “Changes in sports” (*M* = 3.00), while the lowest was for “Individual stressors within the organization” (*M* = 1.91). The skewness and kurtosis values fall within acceptable ranges, suggesting that the variables are approximately normally distributed. All 10 stressor variables are significantly and positively correlated with each other (*p* < 0.001), with correlation coefficients ranging from 0.29 to 0.67. Internal consistency for each variable is strong, with Cronbach’s alpha values ranging from 0.72 to 0.90, indicating that the subscales are reliable.

**Table 3:** Characteristics and correlations of the study variable.

Variable	M	SD	Skewness	Kurtosis	1	2	3	4	5	6	7	8	9	10
1. Athlete’s subordinates’ stressors	2.91	1.07	−0.08	−0.98	(0.90)									
2. Stressors outside of sports	2.81	1.05	0.03	−0.91	0.62***	(0.89)								
3. Individual stressors within the organization	1.91	0.89	1.09	0.41	0.51***	0.53***	(0.88)							
4. Stressors from leader skills	2.18	1.11	0.68	−0.57	0.50***	0.54***	0.60***	(0.85)						
5. Sports conditions	2.25	0.85	0.48	−0.37	0.47***	0.44***	0.29***	0.31***	(0.74)					
6. Team stressors within the organization	2.21	0.92	0.76	−0.00	0.61***	0.60***	0.59***	0.56***	0.43***	(0.77)				
7. Overloading from sports	2.86	1.05	−0.03	−0.88	0.58***	0.57***	0.42***	0.39***	0.54***	0.50***	(0.75)			
8. Changes in sports	3.00	1.06	−0.14	−0.78	0.67***	0.58***	0.45***	0.44***	0.53***	0.48***	0.55***	(0.74)		
9. Sports environment stressors	1.92	0.98	1.00	0.13	0.43***	0.38***	0.56***	0.39***	0.35***	0.42***	0.39***	0.44***	(0.72)	
10. Management stressors within the organization	2.70	1.22	0.11	−1.17	0.67***	0.62***	0.54***	0.56***	0.47***	0.57***	0.60***	0.62***	0.50***	(0.78)

Cronbach alpha values are the diagonal of the correlation table. All vales are significant at the level of 0.001.

**Figure 1:**
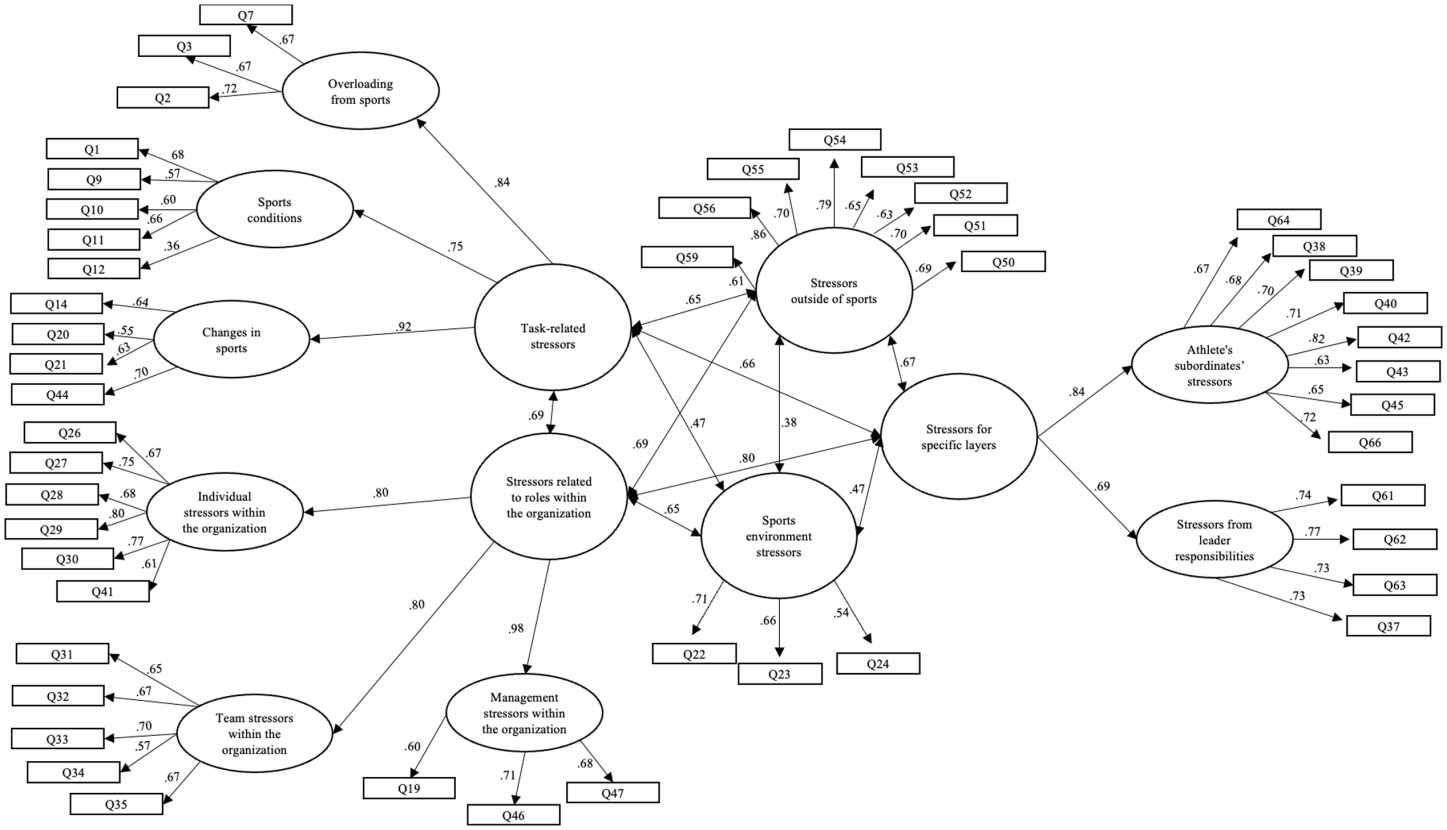
Results of the Structural Equation Model of the work-related stressors. Q = questions; All paths were significantly associated at the level of 0.001.

To test the second-order construct among our factors, we conducted a Structural Equation Modeling (SEM) analysis to evaluate the previously established theoretical model. This model was developed based on the results of our factor analysis. Our findings indicated that the model displayed a good fit with the data and was consistent with established factors (*χ*^2^(1046) = 1572.39, *p* = 0.01; CMIN/d.f. = 1.50; CFI = 0.91; TLI = 0.90; SRMR = 0.06; RMSEA = 0.05). We identified five latent variables as second-order factors, all of which align with the theoretical framework related to sports-related stress. The factors “Task-related stressors” and “Stressors related to roles within the organization” each included three previously established dimensions. Additionally, the latent variable “Stressors for specific layers” comprised two factors. However, we did not create a second-order factor for “Stressors outside of sports” and “Sports environment stressors.” The second-order factors are highly correlated with each other as seen in the subscales of the study. It is important to note that item 59 was moved from “stressors from leader responsibilities” to “Stressors outside of sports “due to its higher reliability and the presence of cross-loadings identified in the factor analysis (Figure 1).

In the next step, the factors were analyzed according to gender and sports variable (Table 4). The analysis revealed significant gender differences across most stress factors, with women consistently reporting higher levels of stress than men. This was particularly evident in “Task-related stressors,” where women (*M* = 3.05, SD = 0.81) scored significantly higher than men (*M* = 2.45, SD = 0.72), *F* = 35.29, *p* < 0.001. Similar patterns were observed for “Overloading from sports” (*F* = 20.71, *p* < 0.001), “Changes in sports” (*F* = 43.92, *p* < 0.001), and “Stressors outside of sports” (*F* = 13.80, *p* < 0.001). In terms of “Stressors for specific layers,” women also reported higher scores in both “Athlete’s subordinates’ stressors” and “stressors from leader responsibilities.” Differences based on sport type (individual vs. team) were also investigated, though generally less pronounced than gender differences. Athletes in team sports reported higher stress in “Task-related stressors” (*F* = 12.08, *p* < 0.001), especially regarding “Changes in sports” (*F* = 24.09, *p* < 0.001), “Overloading from sports” (*F* = 6.18, *p* < 0.05), and “Sports conditions” (*F* = 5.77, *p* < 0.05). Team athletes also experienced greater stress in “stressors from leader responsibilities” (*F* = 7.75, *p* < 0.01) and “Sports environment stressors” (*F* = 14.81, *p* < 0.001).

**Table 4:** Gender and sport type differences between the stress factors.

Variable	Gender			Individual vs Team sports		
	Men M (SD)	Women M (SD)	*F*	Team M (SD)	Individual M (SD)	*F*
**Task-related stressors**	2.45 (0.72)	3.05 (0.81)	35.29***	2.51 (0.77)	2.88 (0.82)	12.08***
Overloading from sports	2.77 (1.09)	3.40 (1.11)	20.71***	2.84 (1.11)	3.19 (1.16)	6.18*
Sports conditions	2.08 (0.77)	2.50 (0.90)	15.21***	2.11 (0.83)	2.37 (0.86)	5.77*
Changes in sports	2.61 (0.95)	3.47 (1.01)	43.92***	2.63 (0.95)	3.28 (1.06)	24.09***
**Stressors related to roles within the organization**	2.15 (0.85)	2.42 (0.85)	5.75*	2.17 (0.81)	2.35 (0.88)	2.66
Individual stressors within the organization	1.87 (0.90)	1.97 (0.96)	0.65	1.80 (0.83)	1.99 (0.99)	2.53
Team stressors within the organization	1.95 (0.89)	2.18 (0.94)	3.93*	2.00 (0.84)	2.09 (0.97)	0.61
Management stressors within the organization	2.49 (1.13)	2.95 (1.30)	8.31**	2.44 (1.13)	2.89 (1.26)	8.40**
**Stressors outside of sports**	2.59 (1.05)	3.10 (1.00)	13.80***	2.75 (1.07)	2.86 (1.05)	0.68
**Sports environment stressors**	1.80 (0.98)	2.07 (0.97)	4.88*	1.65 (0.90)	2.12 (1.00)	14.81***
**Stressors for specific layers**	2.35 (0.91)	2.81 (0.94)	13.62***	2.40 (0.87)	2.66 (1.00)	4.66*
Athlete’s subordinates’ stressors	2.67 (1.03)	3.27 (1.04)	18.38***	2.86 (1.04)	2.98 (1.11)	0.69
Stressors from leader skills	2.04 (1.04)	2.36 (1.18)	4.54*	1.96 (0.98)	2.36 (1.19)	7.75**

*p < 0.05; **p < 0.01; ***p < 0.001. All bold values are significant.

The comparison of stress factors across different sports revealed significant sport-specific differences. As it can be seen in Table 5. The sub factors of “Task-related stressors” significantly differentiated among different sports. Significant differences were found in “Sports conditions” (*F* = 2.38, *p* < 0.05), with the highest stress levels reported by fencers (*M* = 3.49), tennis players (*M* = 3.48), and athletes (*M* = 3.37). Handball (*M* = 2.60) and football players (*M* = 2.75) reported the lowest levels. “Sports conditions” varied significantly as well (*F* = 2.44, *p* < 0.05), with gymnasts (*M* = 2.58) and tennis players (*M* = 2.45) experiencing more stress, while athletes (*M* = 1.98) and ice hockey players (*M* = 1.82) reported less. “Changes in sports” had showed the most pronounced differences across sports (*F* = 4.05, *p* < 0.001). Gymnasts (*M* = 3.33), fencers (*M* = 3.33), and athletes in “other” sports (*M* = 3.22) reported the highest stress levels in this domain, while ice hockey players (*M* = 2.32) and handball players (*M* = 2.55) reported the lowest. “Sports environment stressors” differences were also statistically significant (*F* = 3.10, *p* < 0.01). Tennis players (*M* = 2.39) and basketball players (*M* = 2.17) reported the highest levels of environmental stress, compared to lower levels among handball (*M* = 1.52), football (*M* = 1.56), and ice hockey players (*M* = 1.55).

**Table 5:** Stressor differences between different types of sport.

Variable	Handball M (SD)	Basketball M (SD)	Football M (SD)	Other M (SD)	Fencing M (SD)	Athletics M (SD)	Tennis M (SD)	Ice hockey M (SD)	Gymnastics M (SD)	*F*
**Task-related stressors**	2.43 (0.78)	2.59 (0.94)	2.48 (0.69)	2.82 (0.85)	2.85 (0.52)	2.83 (0.46)	2.86 (0.65)	2.37 (0.77)	2.90 (0.95)	1.77
Overloading from sports	2.60 (1.19)	3.04 (1.35)	2.75 (0.98)	3.18 (1.17)	3.49 (1.00)	3.37 (0.51)	3.48 (0.83)	2.87 (1.20)	2.87 (1.26)	2.38*
Sports conditions	2.21 (0.95)	2.26 (0.73)	2.05 (0.75)	2.30 (0.87)	2.06 (0.74)	1.98 (0.67)	2.45 (0.52)	1.82 (0.82)	2.58 (0.98)	2.44*
Changes in sports	2.55 (0.92)	2.75 (1.04)	2.68 (0.93)	3.22 (1.16)	3.33 (0.57)	3.25 (0.98)	2.97 (1.21)	2.32 (0.81)	3.33 (0.98)	4.05***
**Stressors related to roles within the organization**	2.15 (0.68)	2.40 (0.82)	2.16 (0.79)	2.26 (0.93)	2.51 (0.86)	2.35 (1.02)	2.61 (0.90)	2.04 (0.88)	2.23 (0.81)	0.83
Individual stressors within the organization	1.77 (0.72)	1.98 (0.87)	1.85 (0.84)	1.89 (0.96)	2.09 (0.92)	1.96 (0.93)	2.58 (1.19)	1.57 (0.81)	1.78 (0.84)	1.48
Team stressors within the organization	1.91 (0.67)	2.14 (0.81)	2.01 (0.89)	2.16 (1.04)	2.32 (1.05)	2.04 (1.20)	2.37 (1.01)	1.92 (0.88)	1.80 (0.70)	1.25
Management stressors within the organization	2.53 (1.20)	2.79 (1.25)	2.38 (1.01)	2.61 (1.29)	3.19 (1.26)	2.75 (0.97)	2.88 (1.22)	2.35 (1.20)	2.99 (1.28)	1.19
**Stressors outside of sports**	2.59 (1.01)	2.74 (1.07)	2.89 (1.13)	2.87 (1.09)	2.76 (0.61)	2.57 (1.18)	3.13 (1.21)	2.65 (1.13)	2.76 (0.97)	0.41
**Sports environment stressors**	1.52 (0.65)	2.17 (1.05)	1.56 (0.89)	1.83 (0.95)	2.21 (0.91)	2.15 (1.08)	2.39 (1.03)	1.55 (0.79)	2.18 (1.08)	3.10**
**Stressors for specific layers**	2.53 (0.94)	2.73 (1.02)	2.38 (0.73)	2.52 (1.04)	2.82 (0.95)	2.16 (1.16)	2.91 (0.88)	2.10 (0.88)	2.63 (0.89)	1.42
Athlete’s subordinates’ stressors	2.88 (1.17)	3.22 (1.16)	2.85 (0.99)	2.83 (1.08)	3.07 (0.98)	2.21 (1.41)	3.02 (1.08)	2.52 (1.00)	3.21 (1.01)	1.18
Stressors from leader skills	2.18 (1.15)	2.25 (1.04)	1.93 (0.89)	2.22 (1.17)	2.58 (1.20)	2.18 (1.21)	2.79 (1.20)	1.68 (0.84)	2.06 (1.12)	1.73

*p < 0.05; **p < 0.01; ***p < 0.001. All bold values are significant.

In our final analysis, we analyzed the stress factors across four levels of sport competitions (Table 6). We found only significant differences among “Team stressors within the organization” (*F* = 3.51, *p* < 0.05) and “Stressors outside of sports.” As the post-hoc test showed us National team athletes, (*M* = 2.10), Competing at international level (*M* = 2.09), and competing at national level (*M* = 2.14) reported higher stress than Olympian athletes (*M* = 1.68). “Stressors outside of sports” (*F* = 2.74, *p* < 0.05), showed that National team athletes (*M* = 2.99) and the group of Competing at national level (*M* = 2.91) experienced a higher level of stress in this domain, while Olympian athletes perceived lower levels (*M* = 2.41).

**Table 6:** Stress factor differences among different competition levels.

Variable	Olympian M (SD)	National team athletes M (SD)	Competing at international level M (SD)	Competing at national level M (SD)	*F*
**Task-related stressors**	2.67 (0.96)	2.94 (0.71)	2.70 (0.86)	2.75 (0.84)	0.99
Overloading from sports	2.68 (1.20)	3.27 (1.01)	3.11 (1.20)	3.01 (1.14)	2.15
Sports conditions	2.26 (0.93)	2.45 (0.87)	2.18 (0.90)	2.20 (0.80)	1.09
Changes in sports	3.02 (1.08)	3.22 (0.85)	2.70 (1.20)	3.03 (1.05)	1.82
**Stressors related to roles within the organization**	1.93 (0.72)	2.27 (0.81)	2.28 (0.88)	2.27 (0.90)	2.12
Individual stressors within the organization	1.75 (0.94)	1.81 (0.79)	2.01 (1.01)	1.96 (0.94)	0.87
Team stressors within the organization	1.68 (0.72)	2.10 (0.98)	2.09 (0.91)	2.14 (0.94)	3.51*
Management stressors within the organization	2.40 (1.14)	2.84 (1.21)	2.75 (1.26)	2.71 (1.24)	1.09
**Stressors outside of sports**	2.41 (0.90)	2.99 (0.92)	2.91 (1.20)	2.82 (1.06)	2.74*
**Sports environment stressors**	2.07 (1.00)	2.00 (1.00)	1.86 (0.96)	1.86 (0.99)	0.60
**Stressors for specific layers**	2.44 (0.96)	2.69 (0.82)	2.61 (1.03)	2.50 (0.95)	0.67
Athlete’s subordinates’ stressors	2.88 (1.03)	3.17 (0.89)	2.80 (1.17)	2.89 (1.11)	1.26
Stressors from leader skills	2.01 (1.12)	2.21 (1.15)	2.42 (1.17)	2.11 (1.07)	1.03

*p < 0.05. All bold values are significant.

## Discussion

The goal of this study was to identify work-related factors that may influence athletes’ stress levels. Additionally, we aimed to investigate these stressors across different genders, types of sports, and competition levels. Our analysis identified 10 latent factors that contribute to athletes’ work-related stress and three additional factors that also play a role. Furthermore, we found that female athletes and individual sports participants are more significantly affected by these stressors. However, when examining the differences between sport types and competition levels, only a few significant differences were observed.

Our initial analysis aimed to explore workplace stressors within a population of elite athletes by adapting Juhász’s (2002) general workplace stressor model to a sport-specific context. The results from the factor analysis largely confirmed the applicability of this framework in the athletic domain, with 10 distinct factors emerging. The high internal consistency across all factors (Cronbach’s alpha ranging from 0.72 to 0.90) indicates strong reliability of the adapted scale and supports its use in assessing stress among athletes. Moreover, most of the extracted factors showed strong conceptual alignment with Juhász’s original categories, suggesting that traditional occupational stress models can be effectively transferred to sport environments when appropriately contextualized. For example, factors such as “Task-related stressors,” “Team stressors within the organization,” and “Management stressors within the organization” parallel well-established by Juhász (2002). However, due to its sports context, the names of these constructs were modified for sports settings.

To further examine the structure and coherence of the identified work-related stress factors, we employed Structural Equation Modeling (SEM) to test a second-order theoretical model. This model was grounded in Juhász’s original conceptualization of work-related stress but tailored to the realities of the sport context. The SEM analysis revealed five latent variables that captured broader clusters of stress experiences. Of these, three (“Task-related stressors,” “Stressors related to roles within the organization,” and “Stressors for specific layers”) were successfully modeled as coherent higher-order constructs. In contrast, Stressors outside of sports and Sports environment stressors did not integrate into any higher-order category and remained as independent constructs. Some of the factor labels, such as “Athletes’ subordinates’ stressors” and “Stressors from leader responsibilities,” may not be immediately intuitive in a sports context. These constructs were derived from the adapted Juhász (2002) model and reflect stressors related to interpersonal dynamics and decision-making responsibilities within the athlete’s environment. For example, “Athletes’ subordinates’ stressors” includes items related to peer pressure and expectations from teammates or junior athletes, while “Stressors from leader responsibilities” captures the burden of making career-related decisions or managing team dynamics. Although these terms originate from organizational psychology, we believe they are applicable to elite sport when appropriately contextualized. We believe these factors are a reliable and useful tool for further analysis in sport settings.

We must acknowledge that, there were bidirectional relationships between the construct in our conceptual model. These connections reflect the dynamic interplay between organizational demands and personal identity-based stress. The boundaries between an athlete’s personal life and professional responsibilities are often blurred, as daily training, competition, and organizational expectations frequently spill over into personal domains. For example, role ambiguity or leadership pressure within a team can intensify task-related stress, and external life stressors such as family obligations or relocation may disproportionately affect specific athlete subgroups, including women or younger athletes. This dynamic interplay is supported by recent literature (e.g., Tossici et al., 2024), which highlights the systemic and reciprocal nature of stressors in high-performance environments. Therefore, the bidirectional arrows in our model are not merely visual choices but reflect the theoretical and empirical reality of how stress manifests in elite sport.

Investigating the differences between the stressors and gender we found that female athletes consistently reported higher levels of stress across nearly all domains. This aligns with earlier research showing that women tend to report higher levels of stressful behavior, such as depression, frustration, and anxiety (Asher et al., 2017; Calvarese, 2015). A possible explanation comes from self-construal theory (Cross et al., 2010): women often develop an interdependent self-view, defining themselves through relationships, while men tend toward an independent self-view, focusing on autonomy. It seems a similar pattern can be observed in sport as well, since our results suggest that women might experience the demands of athletic life more intensely or feel less autonomy in navigating those stressors. We believe these results call attention to coaches, sport psychologists, and organizational leaders, who should consider tailoring support programs and resources to meet the specific needs of female athletes.

Investigate different sport types, we found that stress can manifest differently depending on the setting, but for both individual and team athletes. Our results highlighted that individual athletes face more work-related stress than team sports athletes in both of our analyses. We believe the reason behind this phenomenon is that individual athletes may face stress in isolation, while teams typically experience stress collectively (Wergin et al., 2022). The study of Pluhar et al. (2019) shows that athletes in individual sports are more likely to experience anxiety or depression compared to those participating in team sports. Their study attributes this difference to the social and supportive environment of team sports, which fosters enjoyment and stress relief, whereas individual sports often involve a more isolated experience that may increase vulnerability to mental health issues.

Stress levels did not consistently increase with higher levels of competition. In fact, Olympian athletes generally reported lower stress in several domains. This may reflect better coping mechanisms, higher psychological resilience, or more institutional support available at the highest levels (Den Hartigh et al., 2024). These results support the idea that elite experience can be a protective factor for anxiety. Several studies confirm that stress does not increase athletic performance proportionally. For example, the results of Garrido-Muñoz et al. (2024) show that judokas classified as higher-level competitors had better scores on the CD-RISC 10 scale, which measures psychological resilience. This typically indicates a better ability to handle stress and recover from minor or major setbacks. Interestingly, stress levels among national and international athletes were sometimes higher than those of Olympians. This might indicate a “pressure peak” at the sub-elite level, where athletes face intense expectations but have fewer structural supports.

While our study highlights the significance of work-related stress within athletic environments, it also has some limitations that need to be acknowledged. First, we utilized convenience sampling and relied on social media platforms for recruitment, which may have restricted participation. Additionally, the stressors examined were based solely on Juhász’s (2002) framework, potentially limiting the generalizability of our findings and overlooking other constructs that could influence athletes’ work-related stress. Another limitation is that our analysis was confined to Hungarian athletes, which may restrict the generalizability of the findings to athletes from other countries or cultural contexts. Furthermore, our study focused exclusively on a Hungarian sample, making the results applicable only within that context. Future research should address these limitations by incorporating more diverse and international samples, adopting longitudinal designs, integrating mixed methods, and expanding the framework to capture a wider range of individual and contextual influences.

## Conclusion

Our study emphasizes the significance of work-related stress among athletes. Based on our findings, we have the following conclusions: (1) There are 10 factors and three second-order factors for measuring work-related stress among athletes. (2) Women perceive higher levels of work-related stress than men. (3) Individual athletes experience higher levels of work-related stress than those in team sports, as group dynamics play a protective role against anxiety. (4) Work-related stress is not linearly related to competition level. Overall, our research offer several practical implications for the field of sport psychology and athlete support. By identifying 10 distinct stressor categories and modeling them through a theoretically grounded SEM framework, we provide a validated tool that can help coaches, sport psychologists, and organizational leaders better understand the specific stress profiles of elite athletes. Furthermore, our results highlight the need for institutional support, especially for sub-elite athletes who may face high stress with fewer resources. Ultimately, this research enhancing our understanding of sport-specific workplace stress, we can contribute to creating healthier and more sustainable athletic environments.

## Data Availability

The raw data supporting the conclusions of this article will be made available by the authors without undue reservation.
